# ACTIVE - a randomised feasibility trial study protocol of a behavioural intervention to reduce fatigue in women undergoing radiotherapy for early breast cancer: study protocol

**DOI:** 10.1186/s40814-018-0275-7

**Published:** 2018-06-11

**Authors:** N. Courtier, S. Gaze, J. Armes, A. Smith, L. Radley, J. Armytage, M. Simmonds, A. Johnson, T. Gambling, J. Hopkinson

**Affiliations:** 1 School of Healthcare Sciences, Cardiff University, Eastgate House, 35–43 Newport Road, Cardiff, CF24 0AB UK; 2 Faculty of Health and Medical Sciences, School of Health Sciences, University of Surrey, Duke of Kent Building, Guildford, GU2 7XH UK; 3 Centre for Occupational and Health Psychology, Cardiff University, 63 Park Place, Cardiff, CF10 3AS UK; 4 Velindre Cancer Centre, Velindre Road, Whitchurch, Cardiff, CF14 2TL UK

**Keywords:** Cancer-related fatigue, Radiotherapy, Breast cancer, Cognitive behavioural therapy, Randomised controlled feasibility trial

## Abstract

**Background:**

Fatigue is rated as the most distressing side effect of radiotherapy treatment for curable breast cancer. About four in ten women treated experience fatigue, which can last for years after treatment. The impact of this debilitating tiredness is loss of independence and impaired physical and mental function. Our study will take a behavioural intervention with demonstrated effect in treating fatigue in a mixed group of chemotherapy patients and adapt it for women undergoing radiotherapy for early breast cancer. The purpose of this trial is to evaluate the feasibility of delivering the intervention in the radiotherapy pathway for patients at a high risk of fatigue and to explore participants’ experiences of the trial and intervention.

**Methods:**

A pragmatic single-site non-blinded feasibility trial of a behavioural intervention. Main inclusion criteria are prescription of the UK standard 40 Gy in 15 fractions over 3 weeks of radiotherapy (± tumour bed boost) for early (stage 0–IIIa) breast cancer. The total projected sample size after attrition is 70. A previously developed fatigue risk score tool will be used to predict individual’s likelihood of experiencing fatigue. Thirty women predicted to be at a high risk of experiencing significant fatigue will be allocated in the ratio 2:1 to the behavioural intervention or education trial arms, respectively. These feasibility trial participants will be assessed at baseline, after 10 and 15 fractions of radiotherapy and 10 days, 3 weeks and 6 months after radiotherapy. A further 40 women predicted to be at a lower risk of fatigue will join a risk score validation group.

Measures to assess feasibility include recruitment, retention and completion rates and variation in implementation of the intervention. Process evaluation with intervention providers and users includes fidelity and adherence checks and qualitative interviews to understand how changes in behaviour are initiated and sustained.

**Discussion:**

This feasibility study collates data to both inform the progression to and design of a future definitive trial and to refine the intervention.

**Trial registration:**

ISRCTN 10303368. Registered August 2017 (retrospectively registered); Health and Care Research Wales Clinical Research Portfolio (CRP) registration 31419.

## Background

Breast cancer is the most common UK female malignancy with 55,000 cases diagnosed annually [1]. Two thirds of these women will undergo breast conserving surgery followed by adjuvant radiotherapy to prevent loco-regional recurrence. Selective prescription of systemic anti-cancer therapies also contributes to a 5-year survival of 87% for breast cancer [2]. Health policy expects that all survivors of cancer get the support they need to lead active lives [3] and focusses on ‘interventions to help people cope with the side-effects of treatment’ [4]*.*

Patients with early breast cancer rate fatigue as the most distressing side effect of treatment and it is the predominant mediator of wellbeing in this population [5]. Radiotherapy-related fatigue (RRF) disrupts daily functioning—the ability to return to work, to undertake family responsibilities and to maintain social lives [6]—for women who are often of working age (average age of diagnosis is 57 years) [7]. Four in ten women receiving radical radiotherapy experience clinically significant fatigue [8, 9], which can last for months, or years, after treatment [10]. This chronic fatigue is strongly associated with the severity at the end of adjuvant treatment [11].

Whilst results are mixed and pooled effect sizes small to moderate, high-level review evidence reports psycho-educational approaches have demonstrated effectiveness on fatigue reduction (pooled mean effect size − 0.31; range − 0.43 to 1.10, 95%CI − 0.38 to − 0.25) [12]. Effective fatigue interventions are brief, delivered individually to specific disease and treatment groups and based on need [13]. Participants in successful interventions were broadly educated about fatigue, learnt to balance activities and rest, were provided with emotional support and learnt self-management techniques [10]. Cognitive behavioural therapy (CBT)-orientated interventions have shown promise (mean effect size 0.47) [12] in treating fatigue in chronic fatigue syndrome, breast cancer survivors and chemotherapy populations [14]. Goal setting, self-monitoring and feedback are likely to be key techniques for encouraging individual behaviour change [15]. Motivational interviewing (MI) techniques show how a counsellor can use an individual’s concerns and needs as a basis for movement towards a goal [16]. Furthermore, an evidence synthesis by the National Cancer Survivorship Initiative (NCSI) highlights self-efficacy as a key component of effective self-management interventions [17]. The use of physiological feedback, for example a physical activity monitor, can be a powerful tool to promote self-efficacy [18, 19]. The NCSI work also identifies that information alone ‘can increase knowledge and prepare patients for change, and should be provided for all survivors’, but ‘additional tailored support from healthcare professionals’ will be needed for some [17]. Identifying patients who require additional support remains a challenge, but evidence suggests they may be experiencing fatigue and/or anxiety, lacking in a supportive network or have a diagnosis other than invasive ductal carcinoma [20].

The value of education alone, relative to any additional benefit from supportive guidance/contact with a health professional, is unclear when treating cancer-related fatigue. The current intervention has been designed to be consistent with the aspiration of a standardised intervention that is deliverable in a future trial by a range of trained health professionals compared to a fully flexible intervention that may rely on the skills of a professional counsellor: the two interventionists in the current study both have experience of counselling theory and practice. Most cancer-related fatigue studies have been carried out with patients who have developed long-term problems, often from heterogeneous patient groups*.* The aim of the current work is to prevent long-term problems arising within a homogenous population. Of the research conducted around treatment, most involves patients receiving chemotherapy rather than radiotherapy*.* This feasibility study seeks to clarify the preceding uncertainties by testing a psycho-educational behavioural intervention, delivered early in the radiotherapy pathway, to help patients with stage 0–IIIa disease self-manage fatigue.

The *primary aim* of our feasibility study is to evaluate trial processes to determine if the design is feasible and acceptable to deliver in the radiotherapy pathway. The *secondary aim* is to evaluate a participant’s experiences and opinions of the intervention, as a basis for refinement for a definitive trial.

## Methods

### Study design

**Fig. 1:**
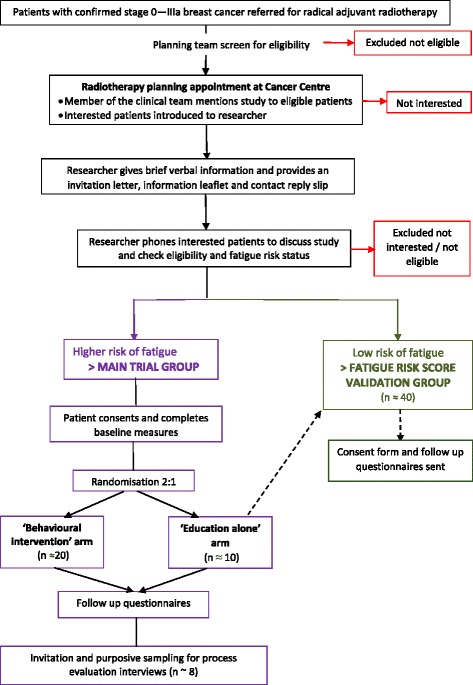
Study schema

A pragmatic parallel-group, randomised feasibility trial (ISRCTN 10303368). Participants will be screened for fatigue risk and dichotomised into low/high risk groups. The higher risk group will comprise the feasibility trial participants, who will be randomly allocated to ‘behavioural intervention’ or ‘education alone’ groups, in the ratio 2:1. Participants at a lower risk of fatigue will join a fatigue risk score validation group: this validation work is required before use in a prospective definitive study. A schema of participant pathways for both groups is shown in Fig. 1.

### Study setting and participant selection

This single-site study will be conducted at a regional cancer centre (referred to as ‘the cancer centre’), which provides non-surgical specialist cancer services to a diverse population of 1.5 million in south east Wales.

### Patient identification

The radiotherapy planning clinical team will screen consecutive patients for initial approach based on the eligibility criteria outlined below. For patients who have not been prescribed adjuvant chemotherapy, screening will be done when the planning lists are compiled using the cancer centre’s electronic patient records system. For patients receiving adjuvant chemotherapy prior to radiotherapy, their medical notes will be screened at their point of consent for radiotherapy (typically chemotherapy cycle four or five of six).

### Inclusion and exclusion criteria

Patients meeting any of the following criteria may be included in the study:

- Females > 16 years;
- Diagnosis of stage 0–IIIA breast carcinoma;
- Standard 4000 cGy in 15 fractions over 3 weeks ± nodal irradiation ± tumour bed boost;
- Able to complete outcome measures.

Patients will be eligible if they have received prior chemotherapy or are receiving monoclonal antibodies or endocrine treatment as maintenance therapy.

If any of the following criteria apply, patients cannot be included in the study:

- Not prescribed radical radiotherapy;
- Concurrent chemotherapy;
- Serious comorbidity causing chronic fatigue;
- Psychiatric illness requiring secondary care intervention;
- Too ill to engage with the intervention in the opinion of the clinical care team.

### Recruitment process

Patients’ usual care involves visiting the cancer centre for a scan from which their radiotherapy treatment is planned. Timings vary, but this typically happens 3 weeks before starting treatment. After their planning scan, a clinical member of the planning team will briefly outline the study to patients. Eligible and willing patients will be introduced to the researcher (SG or NC) and have the opportunity to discuss the study. People who are interested in the study will be given an invitation pack containing a letter of invitation, patient information leaflet, model consent form and a prepaid reply slip (labelled with an anonymised study number.) If a researcher is not available, then a briefed pre-treatment radiographer can give the patient a study pack. Patients can indicate their interest by posting the reply slip (within 5 days) or by choosing on the day to receive a telephone call within 7 days. All patients expressing interest in participating in the study, via face-to-face invitation or the reply slip, will be contacted by telephone to discuss the study. All patients will be given a minimum of 24 h after the initial invitation before being phoned and will have the opportunity to have any questions answered. Study eligibility will be checked and fatigue risk status measured in patients who express a wish to participate.

### Fatigue risk status

Participant risk status will be screened using the Fatigue Risk (Propensity) Score (Fatigue Propensity Score = [3 + (0.13 × fatigue) + (0.16 × anxiety) + 1.1 if diagnosis is not invasive ductal cancer]) [20]. We previously developed this tool to generate a fatigue risk percentage based on pre-treatment factors, as a means to identify patients who are experiencing clinically significant fatigue or are at higher risk of developing fatigue during radiotherapy. The score was generated from study data evaluating variables that contribute to RRF [9]. This data was drawn from 100 women undergoing radiotherapy for breast cancer, none of whom had received prior chemotherapy. Performance of the tool will therefore be evaluated in women who have and have not received chemotherapy. The risk score is weighted towards fatigue level, but the other two items independently add predictive ability. A score of between 3 and 15 is generated. A threshold for high risk can then be applied, which represents a trade-off between sensitivity and specificity, in accordance with clinical judgement or resource constraints [20]. It is anticipated that approximately 50% of patients will score ≥ 5, a threshold that has demonstrated good prediction of patients as fatigued or not fatigued.

- Scoring ≥ 5 will initiate randomisation to one of the feasibility trial arms.
- Participants scoring ≥ 5, who decline entry into the feasibility trial will be offered the opportunity to enter the ‘fatigue risk score validation group’.
- Patients scoring < 5 will join the ‘fatigue risk score validation group’. Participants in this group will be sent a pack containing a consent form and two fatigue questionnaires to be completed at the end of their radiotherapy treatment.

### Sample size

We aim to recruit approximately 77 participants across all study groups. Assuming a 10% loss to follow-up, this will yield final sample sizes of 20, in the intervention group, and approximately 10 and 40 in the control and fatigue risk score validation groups, respectively. These figures are based on logistical grounds and are informed by previous experience in this setting. Assuming a 50% recruitment rate, we would need to approach about 160 eligible women across the 12-month study duration to achieve our planned sample. Recruitment will close when 20 participants have been recruited to the intervention arm.

### Informed consent

The researcher will arrange a mutually convenient date and venue for patients to give consent for the trial. This should typically happen within 10 days of invitation, to enable participants to be allocated to the respective group with sufficient time before their start of radiotherapy. The patient’s written consent will be obtained using the study consent form. Baseline measures will then be completed and the allocation decided via a central randomisation service. The researcher will explain and leave the appropriate study pack with the participant.

### Group allocation

The nature of the intervention means it will not be possible to blind either the researcher or participants to their arm allocation. Analysis of outcome measures will be conducted blind to participant allocation where possible. Randomised group allocation and retention of randomisation codes will be via a central online database service [www.sealedenvelope.com] [21]. This remote allocation service removes the possibility of the researcher influencing group allocation. Allocation to the behavioural intervention arm will be in the ratio 2:1 using a permuted block protocol. A stratification variable will be age (< 57 years and ≥ 57 years). The researcher will inform the psychology team delivering the intervention of patients allocated to the intervention arm as soon as practically possible via a standard referral form. These participants will then be discussed at a psychology team meeting before allocation to one of the two interventionists.

### The behavioural intervention

The intervention was developed by JArm and King’s College London psychology colleagues [14]. Elements are informed by a CBT model of symptom management—the objective being to self-manage fatigue by altering associated thoughts and behaviours. Elements of MI will be incorporated to make sure that individual needs are the starting point to encourage movement towards behavioural goals [16, 17]. The reward (reinforcement) is that in the future the participant may have less fatigue.

The function of the intervention is to motivate helpful behaviour change. The intervention is built on four main components:

- *Education*—written and verbal information about fatigue to increase understanding of fatigue, its causes and consequences. The education content is informed by the Macmillan Cancer Support booklet ‘Coping with Fatigue’ [22];
- *Motivation to change*—the counsellor works to promote self-efficacy and uses persuasive motivational language and assesses the participants’ commitment/readiness to change;
- *Goal setting* and behavioural regulation through self-monitoring of behaviour—to help people plan for and achieve the future goals that are meaningful to the person’s life. These goals may be adjusted with time. All intervention participants will be asked to wear a physical activity monitor, which may be used to set and monitor activity goals according to individual preference;
- *Emotional support*—to help anticipate any negative emotions/barriers (positive language is used to stimulate positive feelings, motivation and importantly enable action.)

​The intervention has been coded by a psychologist using the Behaviour Change Taxonomy (BCT) (http://www.bct-taxonomy.com/), which has been developed to identify the active components of behavioural change interventions [23]. The key components are:

- ​Used a lot throughout: knowledge, goals and behavioural regulation;
- Moderate use of: intentions, reinforcement, emotion;
- Some use of: beliefs about capabilities, optimism, beliefs about consequences;
- And a little use of: professional role and identity and social influences.

The intervention will be delivered on an individual basis. It comprises three 60-min sessions per participant. The sessions (Table 1) will be delivered face-to-face in the psychology department counselling room at the cancer treatment centre and scheduled to coincide with treatment appointments at crucial phases in the treatment pathway: (i) start of week one of radiotherapy, (ii) fatigue intensifies after 10 fractions and (iii) treatment finishes after 15 fractions (or 20 fractions if a tumour-bed boost has been prescribed). One of two professionals attached to the local psychology team (JA and MS) will deliver the sessions. JA is an experienced counsellor and MS is a review radiographer who works holistically with patients during their radiotherapy. A skeleton plan provides some standardisation of session components. Within this structure, the content of the sessions is adaptable to make sure the interaction meets individual needs. Where a patient has routinely completed a holistic needs assessment, this information will help initiate this process of individualisation.

**Table 1:** Summary of intervention components

Time	Content	Behaviour change technique/construct/label
Call 0 After randomisation	Brief scene setting telephone call Introduce counsellor. Prepare for sessions and agree dates for future sessions	
Session 1 Within first few RT treatments	Assessment of current energy state Clarify patterns, causes and impacts of fatigue Identify individual goals and concerns and the meaning of fatigue for this person’s life during RT Assess readiness to change Consider barriers to change *Set three ‘SMART’ goals* • *Activity and sleep diaries and booklet* • Education about fatigue and radiotherapy • Emotional support and encouragement	KNOWLEDGE—of antecedents and health consequences EMOTION—self-assessment of affective consequences BEHAVIOURAL REGULATION—self-monitoring of behaviour BELIEFS ABOUT CAPABILITIES—verbal persuasion to boost self-efficacy Goal setting (outcome and behaviour) Action planning (including implementation intentions
Session 2 Week 2 of radiotherapy	Review of fatigue state Review of diary Guidance with how to manage fatigue and meet goals Prioritisation of activities Best utilisation of upcoming radiography review clinic	GOALS—review of outcome and behaviour goals BEHAVIOUR REGULATION—self-monitoring of behaviour BELIEFS ABOUT CONSEQUENCES—comparative imagining of future outcomes INTENTIONS—commitment GOALS—review of outcome goals Increased self-efficacy, control and positive mood
Session 3 Week 3 of radiotherapy	*Looking to end of treatment and beyond* *Weekly planner and diary review* Modification of activity and goal scheduling *Positivity* *Self-efficacy* *Social support*	BEHAVIOUR REGULATION—self monitoring of behaviour GOALS—review behaviour goals Goal setting—outcome and behaviour goals Encourage self-monitoring and modification of goals; INTENTION—commitment Identification of ambivalent/unhelpful thoughts about end of treatment and beyond; enhancement of approach coping by adopting alternative thoughts Incremental increase in activity levels to achieve goals; normalisation after treatment OPTIMISM—verbal persuasion to boost self-efficacy EMOTION—reduce negative emotions SOCIAL INFLUENCES—social support or encouragement (general)

#### Quality assurance of the behavioural intervention

Intervention training will include the theoretical basis for the intervention and a detailed overview of the manual. The counsellor will record the use of goal setting, activity monitors and any other relevant use of the intervention. Sessions will be audio-taped to enable integrity-to-manual checks. An independent assessor will evaluate fidelity and the extent to which the underlying theory is used for the first three intervention sessions and a random selection of later sessions.

### The ‘education alone’ intervention

Participants allocated to the ‘education alone’ trial arm will be given the Macmillan Cancer Support booklet called *Coping with Fatigue* [22]. This booklet is freely available at information stands within the cancer centre. The content provides information about fatigue and suggests ways of coping with it.

### Fatigue risk score validation group

Participants who are predicted to be at a low risk of fatigue will be allocated to a validation group. These women will fill in a Functional Assessment of Chronic Illness Therapy (FACIT-F) Fatigue Scale [24] on the last day of their treatment and at 10 days and 6 months after their radiotherapy finishes. Any patients estimated to be at a higher risk of fatigue who have declined entry into the feasibility trial will be offered the opportunity to enter the validation group. The fatigue data from the trial control arm participants will also be included. The group data will be correlated to the fatigue risk score calculated before treatment and thereby enable an assessment of the accuracy of the prediction tool. Analysis will be stratified by prior chemotherapy/no chemotherapy. The predictive ability of the tool will be evaluated with a view to potential use in selecting participants in an effectiveness trial.

### Study assessments

#### At screening

The patient’s cancer diagnosis and reasons for ineligibility will be recorded. A screening log will be maintained that records the numbers of people who:

- Are eligible/ineligible;
- Are approached;
- Decline to be contacted and reasons for declining (if proffered);
- Score above and below the fatigue score threshold;
- Decline at the point of consent or trial group allocation.

#### Trial group baseline measures

Baseline outcome measures should be completed directly after consent and prior to random allocation to a trial arm. This will either happen at the cancer centre or the person’s home, depending upon individual preference. The following demographic and clinical data will be recorded on the pre-treatment case report form (CRF) before randomisation:

- Date of birth and postcode;
- Cancer diagnosis and pathological stage and grade;
- Employment status;
- Adjuvant therapies prescribed
- History of previous or concurrent treatments for this cancer—surgery, chemotherapy, endocrine therapy;
- Other relevant medical health history including mental health issues.

#### Trial outcome measures

The feasibility outcomes will include an evaluation of outcome measure completion rates. Outcome measures include Functional Assessment of Chronic Illness Therapy Fatigue Scale (FACIT-F) [24], Hospital Anxiety and Depression Scale (HADS) [25], European Organisation for Research and Treatment of Cancer quality of life physical functioning subscale (EORTC-QLQc30 version 3.0) [26], physical activity as measured by the Fitbit Alta activity tracker and Amy Hoffman’s Self-Efficacy for Managing Chronic Disease scale [27]. The primary endpoint is fatigue as measured by the FACIT-F 10 days after the completion of radiotherapy. Analysis will be stratified by prior chemotherapy/no prior chemotherapy.

The schedule of outcome measures is shown in Table 2. Scale outcome measures will be self-reported by participants (presumed to be at their home). One phone call will be made to prompt a response if outcome measures have not been returned within 7 days of being due. If participants discontinue with intervention, then any completed outcome measurements will be retained for analysis with participant consent.

**Table 2:** Schedule of trial outcome measures

Outcome measure	Pre-RT	Week 2	Week 3	+ 10 days	+ 3 weeks	+ 6 months
		After 10 fractions of RT	After 15 fractions of RT	10 days after RT	3 weeks after RT	6 months after RT
	Baseline	On treatment			Follow-up	
	T0	T1	T2	T3	T4	T5
FACIT-F	X	X	X	X	X	X
HADS	X			X		X
Physical activity		X	X			
EORTC-QLQc30	X		X	X		X
Amy Hoffman’s self-efficacy	X		X	X		

### Trial process evaluations

The feasibility and acceptability of the trial processes will be evaluated with mixed-methods.

#### Evaluation of the feasibility of the trial

- Eligibility rate will be calculated as the proportion of women with stage 0–IIIa breast cancer on radiotherapy clinic lists who also meet the eligibility criteria;
- Recruitment rate will be calculated as the number of people who consent to participate divided by the number of eligible patients approached;
- Eligible patients who decline to enter will be asked to volunteer a reason;
- Retention rate will be calculated as the number of participants who complete all outcome measures divided by the number who record baseline outcome measures;
- Adherence to the intervention will be monitored by recording the number of sessions completed;
- Reasons for discontinuation or non-adherence should be sought, where possible.

The definitive trial will be considered feasible if > 70% of participants complete all the interventions and outcome measures. If this rate is between 65 and 70%, then adjustments in future work should be considered. A rate < 65% requires substantive change to the intervention and/or to the trial process.

#### Acceptability

Patient participant’s and interventionists’ views on the acceptability of intervention and trial processes will be captured in semi-structured telephone interviews. All interviews will be recorded with the interviewee’s permission. Questions to understand if, and how, changes in thoughts or behaviour are initiated and sustained will be based upon the active ingredients identified at coding theory of BCT. Analysis will be inductive, with new themes being incorporated into subsequent interviews.

Interviews will be conducted with about eight intervention patients. The aim is to conduct participant interviews within 2 months of the end of their radiotherapy sessions. A sample will be chosen that reflects maximum variation in compliance with and response to the intervention. Interviews will also be conducted with two control group participants to explore acceptability of trial processes, any impacts of participation and use of the educational booklet. Interviews will last up to 40 min and will be stopped if the interviewee becomes too fatigued or distressed.

This enquiry will be supplemented by an in-depth interview with both of the professionals delivering the intervention. These semi-structured interviews will explore challenges to delivery, perceived successes, barriers to implementation/suggestions to improve intervention processes and opinion of whether the intervention was delivered as intended. The perceived value of the intervention and factors that promote or inhibit patient adherence and integration into daily life will be explored.

### Data analysis

The study can be considered as the modelling phase of the Medical Research Council guidelines for developing complex interventions [28]. As a feasibility study, the purpose is not to make a formal analysis of the primary outcome. The purpose is evaluation of trial processes to determine whether to progress to a study of effectiveness and to estimate unknown parameters needed to design this study.

Outcome measure data will be analysed blind to arm allocation where possible. Feasibility descriptive data will evaluate eligibility, recruitment and retention rates and acceptability of and adherence to the intervention with mean/median point statistics and 95% confidence intervals. Changes over time of the outcome measures will be described by group. Adherence rates according to baseline measures may be analysed to assess the role of mediators, such as prior chemotherapy prescription.

All interviews will be recorded and transcribed. Data analysis will be performed using a framework approach [29]. A coding framework for emergent themes will be developed, validated and compared. Twenty percent of the data will be double-coded by a second researcher to check reliability of coding to enhance rigour.

### Ethical considerations

The study was approved by a local NHS research ethics committee in August 2016 (16/WA/0205) and will be conducted in accordance with the principles of Good Clinical Practice (GCP). The ethical permission granted permission to collect reasons as to why women decline to participate to inform the evaluation of feasibility of the study. The study has also been approved by the local National Health Service Research and Development committee.

### Safety monitoring

All potentially serious adverse events related to the behavioural intervention will be reported to the principal investigator within 24 h of the team being aware of its occurrence. The psychology team will consider each referral to the intervention delivery team at a team meeting, in line with the cancer centre’s standard operating procedures. This will include a consideration of any known support that may have been sought or offered (internally or externally to the cancer centre) for psychological concerns. Participants will be informed if they score > 19 on the HADS and the option of notifying their GP will be discussed with them.

### Dissemination policy

This protocol (version 2.0, date 14 December 2016) has been reported in line with SPIRIT guidance [30]. The findings will first be reported to the funders, then communicated to participants. At least one open access publication and conference presentation will disseminate results to relevant health professionals within 12 months of the trial closing. All members of the overseeing trial advisory group (TAG) will be invited to be co-authors.

## Discussion

Fatigue is the biggest patient concern before, during and after radiotherapy for women with early breast cancer [31]. Our group’s previous exploratory and observational work indicates that approximately 60% of women experience mild fatigue that does not greatly disrupt lives [9]. However, we have also used our Fatigue Risk Score to evidence a distinct patient group who are at high risk of fatigue before radiotherapy [20]. Many factors can contribute to fatigue, but women with raised levels of perceived stress before treatment are most at risk from later behavioural problems like fatigue [9, 32]. Furthermore, maintenance and pacing of activity is important, but the short-term adoption of vigorous exercise is likely to be counterproductive for patients at a high risk of fatigue.

The current study builds on the preceding developmental work by refining and testing an intervention to reduce fatigue. This intervention is novel because it is instigated early in the radiotherapy pathway to target the women who are most likely to benefit (any reduction in fatigue for low-risk women is likely to be very small). The aim is to support coping using behavioural techniques. A strength of the study is the homogeneity of the participants’ disease and treatment characteristics. Although appropriate outcome measures to test for effects are being used as part of the study protocol, the trial is neither designed nor powered for formal analysis of effectiveness. The purpose is to address the current uncertainties in optimal trial and intervention design to inform a future definitive trial.

## Abbreviations

BCT: Behaviour Change Taxonomy
CBT: Cognitive behavioural therapy
cGy: Centigray (unit of absorbed radiation)
CRF: Case report form
EORTC-QLQc30: European Organisation for Research and Treatment of Cancer quality of life physical functioning subscale
FACIT-F: Functional Assessment of Chronic Illness Therapy Fatigue Scale
GP: General practitioner
HADS: Hospital Anxiety and Depression Scale
ISRCTN: International Standard Randomised Controlled Trials Number
MI: Motivational interviewing
NCSI: National Cancer Survivorship Initiative
NHS: National Health Service
REC: Research Ethics Committee
RRF: Radiotherapy-related fatigue
TAG: Trial advisory group
